# What influences individual perception of health? Using machine learning to disentangle self-perceived health

**DOI:** 10.1016/j.ssmph.2021.100996

**Published:** 2021-12-09

**Authors:** Jordi Gumà

**Affiliations:** Department of Political and Social Sciences (Universitat Pompeu Fabra), Spain

**Keywords:** Self-perceived health, Machine learning, SHARE survey, Health outcomes, European health, European population

## Abstract

Self-perceived health is a subjective health outcome that summarizes all the health conditions and is widely used in population health studies. Yet, despite its well-known relationship with survival, it is still unclear as to which health conditions are actually taken into account when making an individual assessment of one's own health. The aim of this paper is to assess the influence of four objective health conditions – IADLs, ADLs, chronic diseases, and depression – in predicting self-perceived health among Europeans by age group (50–64 and 65–79) and by sex.

Classification trees (J48 algorithm), which pertains to the emerging Machine Learning techniques, were applied to predict self-perceived health according to the four abovementioned objective health conditions of European individuals in the sixth wave of the Survey of Health, Ageing and Retirement in Europe (SHARE) (n = 55,611).

The four variables present different degrees of relevance in establishing predictions of self-perceived health values by age and by sex. Before the age of 65, chronic diseases have the greatest importance, while IADL limitations are more important in the 65–79 age group. Likewise, ADL limitations are more important for women free of chronic diseases in the 50–64 age group; however, these differences disappear among women in the older group.

There is an evident degree of interplay between the objective health indicators of chronic diseases, ADLs, IADLs, and depression when predicting self-perceived health with a high level of accuracy. This interplay implies that self-perceived health summarizes different health conditions depending on age. Gender differences are only evident for the younger age group, whereas construction of self-perceived is the same for women and men among the older group. Therefore, none of these four indicators on its own is able to totally substitute self-perceived health.

## Highlights

• Self-perceived health constitutes a black box because of its subjective nature and the fact that it summarizes all the health conditions of an individual in a single indicator.
• This is one of the first studies to apply predictive algorithms, as developed in machine learning, to an examination of population health.
• Results in this study provide valuable insights into what self-perceived health actually encompasses.
• Future work using self-perceived health will benefit from a much more accurate understanding of this health indicator.

## 1 Introduction

The increasing availability of individual health data has made it possible to obtain more detailed information about the health of the population in the world's most developed countries. Moreover, the current diversity of health measures enables us to capture the multidimensionality of the population's health as defined by the World Health Organization (1948). However, despite the diversity of health indicators, there is still a need to use unique indicators to summarize a population's health in a standardized fashion as has traditionally been the case with mortality. Among such measures, self-perceived health stands out as being an indicator that is based on an individual's declaration of their own state of general health, without their having to justify their choice of any given response. In the academic community, there is, and has been, considerable interest in evaluating its actual capacity for approximating the objective health profile of a population. Indeed, while subjective health seems to predict the short- and medium-term survival of individuals in both adult (Singh-Manoux et al., 2006; Tamayo-Fonseca et al., 2013) and old ages (Quesnel–Vallée, 2007), a number of studies conducted on populations at more advanced ages have shown that the association between these two indicators decreases with increasing age (Franks et al., 2003; Zajacova & Woo, 2016), thus weakening the general belief that self-perceived health and mortality show parallel patterns across all age groups.

Self-perceived health, therefore, constitutes a black box precisely because of its subjective nature and the fact that it summarizes all the health conditions of an individual in a single indicator. The subjective nature of this indicator lies, at least partially, in the influence that the context can have on the individual's response, as pointed out by Jylhä (2009). The author pointed three key contexts for their influence on the perception of individuals: the individual biography of health; the reference groups taken for this evaluation (how I am in relation to my peers); and the cultural conventions that condition the response within the measurement scale. Thus, although a deterioration in health measured on the basis of this indicator has the capacity to anticipate an increase in the individual's probability of dying (DeSalvo et al., 2006; Idler & Benyamini, 1997), we still lack exhaustive knowledge as to which health conditions are actually being taken into consideration when an individual undertakes an assessment of their own health. There are precedents, however, that have assessed the relationship between self-perceived health and specific aspects of health such as chronic conditions (Malmusi, Artazcoz, Benach, & Borrell, 2012), functional health (Golini & Egidi, 2016) and depression (Kosloski et al., 2005); yet, the link between self-perceived health and the set of possible objective health conditions of an individuals has, to date, not been fully established. In this line, recent research by Lisko et al. (2020) on the structure of self-perceived health for the specific case of the nonagenarian population in Finland showed how some health conditions such as fatigue, depression, problems in mobility, dizziness, deficits in vision and heart disease were directly associated with poor SRH.

The aim of this short communication is to assess the influence of objective health conditions, both physical (IADLs, ADLs, chronic diseases) and mental (depression), in predicting responses to questions about self-perceived health among European women and men between the ages of 50 and 79. The health conditions that contribute to a greater predictive capacity of self-perceived health should be those that have the greatest influence on an individual's perception of health. For this purpose, classification trees employed in machine learning, namely the J48 algorithm (updated version of the C4.5 algorithm), are applied to European individuals who participated in the sixth wave of the Survey of Health, Ageing and Retirement in Europe (SHARE) (Börsch-Supan, 2013). This classification tree algorithm was specifically chosen as it provides a graphical representation of the relationship between variables in predicting self-perceived health. This representation provides information both on the degree of importance of each variable and on the interaction between them for making the final prediction.

The working sample was divided into two different age groups (50–64 and 65–79) in order to account for the effect of age on the influence of objective health conditions on self-perception of health, with older individuals showing a more favorable health perception (Shields & Shooshtari, 2001). The age boundary of 79 was fixed because this has been shown to be the age at which the relationship between mortality and self-perceived health begins to weaken (Zajacova & Woo, 2016). In addition, analyzing two age groups separately also aims to take into account that individuals take their peers as a reference to evaluate their own health, as stated by Jylhä (2009). The two independent models explore whether there are differences in the health conditions that have an influence on self-perceived health. Furthermore, given the gender gap in self-perceived health – with women declaring higher prevalences of poor health (Oksuzyan et al., 2018) – all calculations were made separately for women and men.

The contribution is twofold: first, this is one of the first studies to apply predictive algorithms, as developed in machine learning, to an examination of population health; and, second, thanks to the exploitation of this particular methodology, the evaluation I undertake of the contribution of objective health situations to individual (both female and male) perceptions of health provides valuable insights into what self-perceived health actually encompasses. This complements the recent research on the structure of this subjective health outcome by Lisko et al. (2020) using a more flexible nonparametric method such as classification trees (see Buskirk and Kirchner, 2020- for further details).

## 2 Material and methods

The data analyzed herein are taken from the sixth edition of the Survey of Health, Ageing and Retirement in Europe (SHARE). SHARE is a panel survey that is representative of the non-institutionalized population in Europe, aged 50 and over. It gathers information about multiple aspects of this population, including demographics, work, family, health, housing, etc (Börsch-Supan, 2013). The interviews for this sixth edition of SHARE were conducted in 2015. Although two more waves of the survey have subsequently been conducted, this is the most recent edition of the survey in which all questions included in the general panel survey questionnaire were comprehensively asked to all survey participants (note, seventh wave respondents that did not participate in the third wave responded to a different questionnaire which included biographic questions) and in which the respondents were not influenced by the COVID-19 pandemic (note, the eight wave was conducted in the first quarter of 2020, coinciding with the outbreak of the pandemic). The different analyses were conducted based on a single edition of the survey since, being a panel survey, the same persons could be part of the test or validation data set on several occasions, although at different ages.

The working sample comprised all persons aged between 50 and 79 years old residing in the 17 European countries (Israel was discarded from the analysis) participating in the sixth edition of SHARE who answered all the questions included in our analysis (n = 53,664 from an original sample of 55,611, 96.5% response rate). All the variables taken into consideration to train the classification algorithms were dichotomized so as to facilitate the interpretation of the resulting classification trees. Previous trials employing just three categories for all the health outcomes other than self-perceived health had resulted in confusing classification trees and no meaningful improvements in their accuracy (less than 3 percentage points on average). The variables considered were the following:

- Number of chronic diseases. This variable is the result of aggregating responses to the battery of questions as to whether the person interviewed has been diagnosed with any of the all possible chronic diseases included in the questionnaire (18 in total). The final variable was: no chronic disease; one or more chronic diseases.
- Instrumental activities of daily living (IADL). Participants were asked whether they had any difficulty doing each of the following everyday activities: doing work around the house or garden; leaving the house independently/accessing transportation; shopping for groceries; doing personal laundry; managing money; preparing a hot meal; taking medications; and making telephone calls. Individuals were required to exclude any difficulties expected to last less than three months. The final variable was: no limitations; being limited to perform one or more activities.
- Activities of daily living (ADL): Participants were asked whether, “because of physical, mental, emotional, or memory problems”, they had any difficulty doing these activities (again, excluding any difficulties expected to last less than three months): dressing (including putting on shoes and socks); eating (such as cutting up your food); using the toilet (including getting up and down); bathing and showering; getting in and out of bed; and walking across a room. The final variable was grouped as follows: no limitations; being limited to perform one or more activities.
- Depression: This variable was measured using the EURO-D scale, developed and validated by the EURODEP Concerted Action Project (Prince et al., 1999). EURO-D compiles binary information about 12 different symptoms of depressive moods: depression, pessimism, wishing death, guilt, sleep, interest, irritability, appetite, fatigue, concentration, enjoyment, and tearfulness (Prince et al., 1999). The scale ranges from 0 to 12 with a score above 3 representing significant depression levels (Castro-Costa et al., 2008). Consequently, the categories for the final variable were: not depressed (values of 3 or lower); depressed (values above 3).
- Self-perceived health: This variable was based on responses to the question ‘How is your health in general?’ (excellent; very good; good; fair; poor). Following common practice (Croezen et al., 2016), we grouped the five possible answers into two categories: excellent, very good or good health (good health = 0), and fair or poor health (poor health = 1).

The classification algorithm selected was the J48 (Witten & Frank, 2002), an updated version of the algorithm C4.5 proposed by Quinlan (2014). This algorithm belongs to the group of classification trees (Loh, 2011) whose objective is to use the information to learn how different variables are hierarchically related in order to understand the decision-making process or to highlight a certain characteristic. In the case of decision trees defined by categorical variables – as is the case here – the relationships between the different nodes in the classification tree are established logically by answering the question as to whether a respondent presents a certain characteristic or not. The decision tree is constructed by starting with the variable with the greatest capacity to discriminate the final classification. This establishes the root node. Taking this variable, the decision tree can then be branched to show all possible routes, thus illustrating the interrelation between variables and resulting in the prediction of one outcome or another. The order of the variables within each of the branches informs about the relative importance of each attribute to predict the outcome until the algorithm manages to reach a prediction that can be considered reliable.

The k-fold cross-validation procedure was used in order to avoid the problem of overfitting, i.e. when the noise in the training data has a relatively high influence on the learning process of the model (Kohavi, 1995^)^. Under k-fold cross-validation the data are randomly partitioned into k different subsets of approximately equal size. In the ith fold of the cross-validation procedure, the ith subset is used to test the performance of a model trained on the remaining k − 1 subsets. The average of the performance observed over all k folds allows us to estimate the performance of a model trained on the entire sample (Cawley & Talbot, 2010). In this case, the number of folds was set at 10.

Studies of the relationship between self-perceived health and mortality have shown this relationship to be age-dependent, becoming less intense as population groups in older ages are studied (Zajacova & Woo, 2016). For this reason, we opted to estimate independent models for two distinct age groups (50–64: Pre-retirement age; 65–79: Post-retirement age). In this way, we also wanted to determine whether the factors affecting self-perceived health differ according to age as a consequence of the differences in the health profile according to the variables analyzed (see Table S1 for further details). In addition, we also calculated different classification trees for women and men due to sex differences in health, such as the male-female health survival paradox (Oksuzyan et al., 2018).

The predictive capacity of the J48 algorithm was validated by comparing the accuracies (% of successes) of each of the models obtained using this algorithm with the values obtained from the random forest classification tree, an algorithm that fits an ensemble of decision tree models and calculates the average between all possible decision trees (Breiman, 2001). The latter was chosen to validate the results on the grounds that calculating the average between several decision trees has been shown to minimize possible classification errors due to the fact that it focuses on a single tree (Cutler et al., 2012). For the two age groups, the accuracy of the J48 model was almost identical to that of the random forest results for both women and men: 75.8 and 76.9% in the first age group, respectively, and 71.4 and 72.1%, respectively, for the second age group when using J48 vs 75.9 and 76.9%, respectively, in the first age group, and 71.5 and 72.0%, respectively, in the second age group when using the random forest algorithm. All the models and their respective measures of accuracy were calculated using the Waikato Environment for Knowledge Analysis (WEKA) (Frank et al., 2009) software version 3.8.5.

## 3 Results

**Fig. 1:**
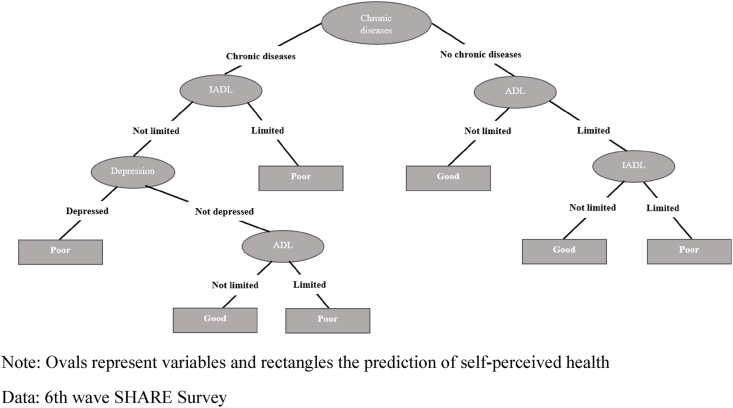
Decision tree for self-perceived health. Age group 50–64. Women. Note: Ovals represent variables and rectangles the prediction of self-perceived health Data: 6th wave SHARE Survey.

**Fig. 2:**
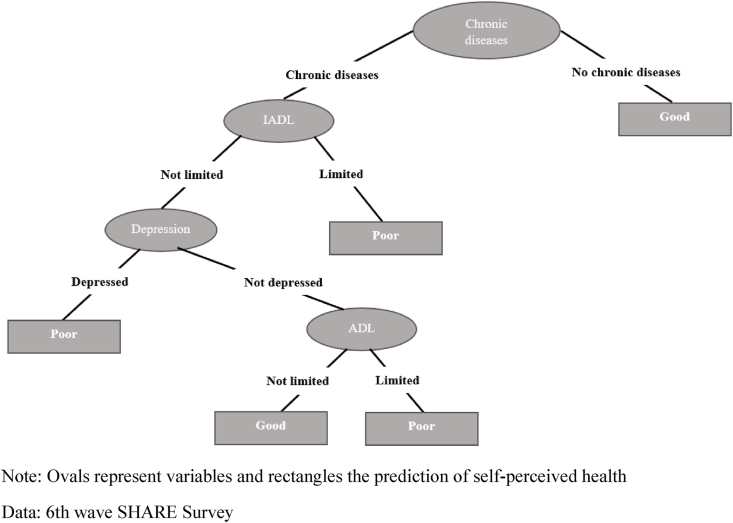
Decision tree for self-perceived health. Age group 50–64. Men. Note: Ovals represent variables and rectangles the prediction of self-perceived health Data: 6th wave SHARE Survey.

Fig. 1, Fig. 2 show the resulting decision trees for women and men, respectively, in the 50–64 age group. In the case of women, chronic diseases constitute the root node, being the variable with the greatest capacity to predict self-perceived health. The left branch corresponds to those reporting a chronic disease whereas the right branch represents those reporting no chronic disease. This left branch comprises three additional nodes, defined by IADL limitations, depression and ADL limitations. These three nodes provide a prediction of poor health in those cases in which an individual reports presenting one of these health conditions (i.e. an IADL limitation, depression or an ADL limitation), whereas when an individual reports not presenting the corresponding health condition, a new node is opened culminating in the final node, represented by ADL limitations. Note, that in the case of this left branch, only those that reach the end of the tree without reporting any IADL limitations, depression or ADL limitations are predicted to have good self-perceived health. In contrast, the right branch, corresponding to those without any chronic disease, comprises just two additional nodes defined by ADL and IADL limitations. In this case, only those with both types of limitation are predicted as presenting poor health whereas all other respondents are predicted to be in good health.

Fig. 2 shows that the male pattern for the 50–64 age group is the same as that presented by their female counterparts when both report a chronic disease (left branch). The main difference occurs in the right branch (respondents without a chronic disease) which ends directly in good health without any additional node being opened.

**Fig. 3:**
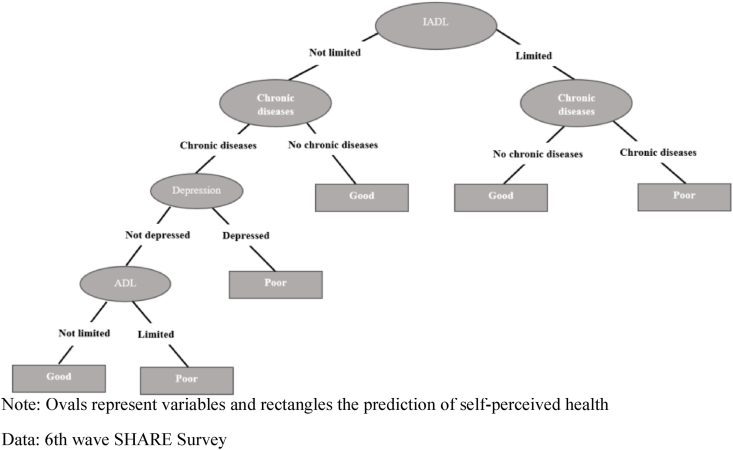
**Decision tree for self-perceived health. Age group 65**–**74. Women**. Note: Ovals represent variables and rectangles the prediction of self-perceived health Data: 6th wave SHARE Survey.

**Fig. 4:**
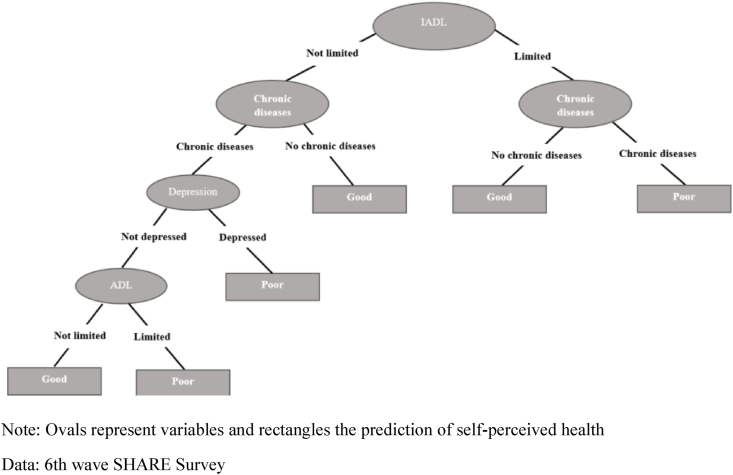
Decision tree for self-perceived health. Age group 65–74. Men. Note: Ovals represent variables and rectangles the prediction of self-perceived health Data: 6th wave SHARE Survey.

If we examine the classification trees for women and men aged between 65 and 79 (Fig. 3, Fig. 4, respectively), we observe how both sexes now display exactly the same pattern for self-perceived health. Unlike the pattern presented in the younger age group, the first node for both sexes is defined by IADL limitations and a more complex left branch for those without these limitations that includes all the other health conditions. In these trees, both the left and the right branches have a second node defined by chronic diseases, although this is the last node on the right branch, with those reporting a chronic disease predicted to be in poor health and those reporting to be without a chronic disease predicted to be in good health. In the case of the left branch, for those who do not report any IADL limitations, the fact of their having a chronic disease is not decisive for predicting their self-perceived health, whereas not having one of these diseases is always a prediction of being in good health. However, those individuals reporting a chronic disease subsequently present a third and a fourth node defined by depression and ADL limitations, respectively. The fact of presenting depression is a prediction of poor health, whereas those that do not present depression continue along the classification tree to the last node defined by ADL limitations, with those presenting one of these limitations predicted as being in poor health and those not presenting any limitations predicted to be in good health.

## 4 Discussion

This study assesses the influence of various objective health conditions, both physical (IADLs, ADLs, chronic diseases) and mental (depression), in predicting responses about self-perceived health in European women and men aged between 50 and 79. The predictive capacity was assessed by means of classification trees based on the J48 algorithm, which belongs to the family of machine learning methods, and by using data from the sixth wave of the SHARE survey. The approach reported contributes to a better understanding of a health outcome that has been used to describe general population health profiles without our having a clear idea of what health conditions actually influence individual perceptions. The results complement and expand recent research on health situations associated with self-perceived health (Lazarevič & Brandt, 2020; Lisko et al., 2020) by using a more flexible nonparametric method such as classification trees.

The results reported herein confirm the high predictive capacity of self-perceived health based on four of the most widely used health indicators in the literature, namely chronic diseases, IADLs, ADLs and depression. However, differences are observed between the two age groups (50–64 and 65–79) in terms of the order of importance of each of these four variables in establishing predictions of self-perceived health values. Before the age of 65, chronic diseases are shown to have the greatest importance in predicting the self-perceived health of people in this age group, while among those over the age of 65, IADL limitations become more important. This shift in the influence attributable to different health situations seems to be in line with the effects of the disablement process proposed by Verbrugge and Jette (1994), which is initiated by pathologies, which subsequently give rise to impairments followed by functional limitations. In fact, the disablement process would be one of the contextual references that individuals would take to self-declare their health, as pointed out by Jylhä (2009). The perception of whether one is in an early, advanced or middle stage for one's age would lead to establish the decision sequence about one's own health status. It should be noted that the predictive capacity of chronic diseases loses prominence as these diseases become more prevalent among the population (see Table S1). This result is in line with findings of Lazarevič and Brandt (2020) who observed that the contribution of diseases to explain the variability of self-perceived health decreased with age, while that of functioning increased. What is observed in both age groups is the complementary role that depression and ADL limitations play in predicting self-perceived health when this cannot be established directly from IADL limitations and chronic diseases.

The differences between the sexes, as regards the contribution of the four health indicators used in defining the classification trees, are found to be less notable in the older group. This would confirm the non-existence of a gendered pattern of response to the question on self-perceived health at least at older ages, as previously pointed out by Oksuzyan et al. (2019). This similarity in the influence of the four health conditions reinforces the belief that the differences in health between women and men that emerge in the gender paradox are due to the more unfavorable female profile in terms of health and its sociodemographic determinants (Crimmins et al., 2011; Author). However, important differences are observed in the age group before the age of 65. Among men in this age group, not presenting a chronic disease leads directly to a prediction of good health, whereas among women the situation is more complex and is conditioned by their limitations in carrying out certain activities. In fact, it is noteworthy that among women without chronic diseases, ADL limitations show a greater predictive importance than those in IADLs, this being the only case in which this sequence is observed in the explanatory order.

## 5 Conclusions

All in all, this study has demonstrated the complementary nature of the four health indicators analyzed (chronic diseases, ADLs, IADLs and depression) in predicting self-perceived health with a high level of accuracy. This complementarity implies, however, that none of these four indicators on its own is able to substitute self-perceived health as a general indicator of health. This conclusion is strengthened by the fact that the predictive capacity of the four health indicators of self-perceived health has been shown to vary according to the age group analyzed, and even according to sex among those under the age of 65. Therefore, researchers should be aware of what self-perceived health actually encompasses when taking it as an indicator to capture differences and inequalities in health for the general population. Future research needs to explore whether this pattern of prediction differs across countries or education attainment, in line with the differences in health perception across European countries or education reported in earlier studies (Eikemo et al., 2008; Author, 2019; Lazarevič & Brandt, 2020).

## Author statement

Jordi Gumà: Conceptualization, Methodology, Data curation, Writing- Original draft preparation.

## Ethical statement

The University of Mannheim's internal review board reviewed and approved SHARE for wave 1 to 4. Ethics Council of the Max Planck Society conduct the ethics reviews for wave 4 onwards. Ethics approvals in each of SHARE-participating countries were obtained from the local ethics committees or institutional review boards. SHARE data is available for scientific community and may only be used for scientific research. The secondary data analysis of SHARE data, such in the present study, do not require further ethical approval. Details on SHARE ethical approval is available in http://www.share-project.org/fileadmin/pdf_documentation/SHARE_ethics_approvals.pdf.

## Funding

This work was supported by the FEDER/Spanish Ministry of Science, Innovation and University/Spanish Agency of Research and is part of the project “Prevention is better than cure when ageing is behind the door: interplay between social determinants of health in Spain (INTERSOC-HEALTH)” (RTI2018-099875-J-I00 -MCIU/AEI/FEDER, UE- PI: Jordi Gumà).

## Declaration of competing interest

The authors report no conflict of interest.
