# Supplementary material for: What influences individual perception of health? Using machine learning to disentangle self-perceived health
Source: SSM Popul Health. 2021 Dec 9;16:100996. doi: 10.1016/j.ssmph.2021.100996 (PMC8669356; doi:10.1016/j.ssmph.2021.100996)
Supplement: Multimedia component 1 [file mmc1.docx]

**Table S1. Predictive importance of each attribute in the classification algorithm in establishing predictions of self-perceived health values by age and by sex.**

|  | **50-64** | | **65-74** | |
| --- | --- | --- | --- | --- |
|  | **Importance** | **Attribute** | **Importance** | **Attribute** |
| **Women** | 0.12 | Chronic diseases | 0.09 | Chronic diseases |
|  | 0.09 | IADL | 0.09 | IADL |
|  | 0.07 | Depression | 0.08 | Depression |
|  | 0.06 | ADL | 0.05 | ADL |
| **Men** | 0.14 | Chronic diseases | 0.10 | Chronic diseases |
|  | 0.10 | IADL | 0.08 | IADL |
|  | 0.06 | Depression | 0.07 | Depression |
|  | 0.05 | ADL | 0.04 | ADL |

Source: 6^th^ wave the SHARE survey
